# Expandable Micro-motor Bur, design of a new device for least invasive extraction of broken teeth roots

**DOI:** 10.1186/1750-1164-7-2

**Published:** 2013-03-05

**Authors:** Amir Hashem Shahidi Bonjar

**Affiliations:** 1 Students Research Committee, Dental School, Shahid Beheshti University of Medical Sciences, Tehran, 1983963113, Iran

**Keywords:** Tooth extraction, Complicated root extraction, Expandable micro-motor bur, EMB

## Abstract

**Background:**

Extraction of a broken tooth root is often a traumatic experience for both the practitioner and the patient. To extract broken roots, generally invasive approaches as open window surgeries or mucoperiosteal flap and/or removal of buccal bone are performed.

**Presentation of the hypothesis:**

Expandable micro-motor bur (EMB) is a hypothetical design of a dental instrument proposed for removal of broken teeth roots that cannot be extracted by the routine closed methods and in which common instrumentations cannot afford to accomplish. Implication of EMB would introduce a new technique in removal of broken teeth roots in which surgical trauma is minimized and so post-extraction disorders. It would eliminate surgical invasion to the surrounding tissues; and also it would eliminate profound hand forces by the practitioner, consequently reduces stress for both the practitioner and the patient. It would eliminate high risk aftermaths such as operative morbidity (due to bone loss), maxillary sinus exposure and probable need for additional surgery as are indicative of some conventional open access approaches.

**Testing the hypothesis:**

Further studies are needed to confirm its effect in clinical cases. The effectiveness of EMB should be verified firstly by animal experiments. The likelihood of its negative influence on nearby vascular and nerve system should be well evaluated.

**Implications of the hypothesis:**

Implication of EMB would be of interest to both patients and the surgeon due to the following main achievements: a) no need for mucoperiosteal flap, hence preservation of soft tissue, b) no need for osteotomy, hence retention of buccal bone, c) less risk of sinus exposure, d) minimum chance of post operative infections due to eliminated surgeries in soft tissues and bones and e) in terms of esthetics, it will have a special meaning for immediate placement of dental implants. EMB’s structural components include Bur head, Spacers and Bur base. A micro motor would power its spin. In contrast to conventional surgical approaches, EMB procedure is conservative. It is anticipated that EMB would provide less traumatic and least post-operative complications in extraction of broken teeth roots.

## Background

The broken teeth roots may be difficult to remove and the dentist should strongly consider performing an open extraction after initial attempts at forceps removal have failed [1-3]. Open extractions include invasive open window surgeries or mucoperiosteal flap and/or removal of buccal bone [4-10]. Occasionally, it is necessary to prepare a purchase point with the bur and to use an elevator as the Crane pick to elevate the remaining root [11,12]. However, these treatments have aftermaths characterized by post-operative disorders [13-17]. Therefore, seeking an easy and effective method to remove broken teeth roots and resolve the operative and post operative complications is necessary for dental clinicians and the patients.

### General information on extractions of broken teeth roots

**Figure 1:**
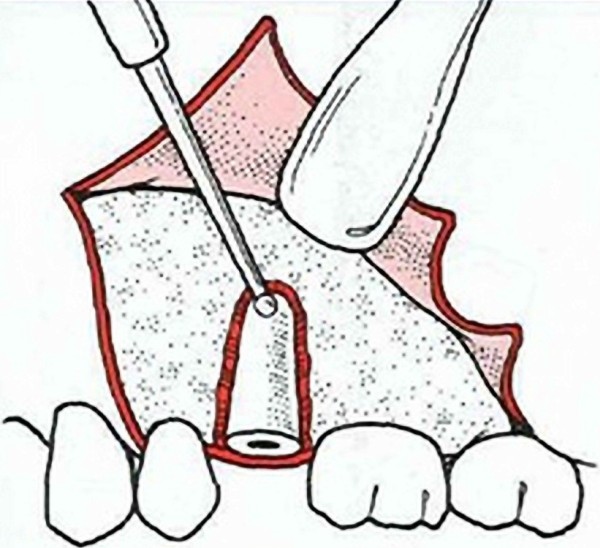
**A conventional open root extraction surgery **[1]. The sketch shows soft tissue flap and removal of buccal bone for insertion of elevator to elevate the root from its socket. This invasive approach may cause more operative morbidity due to bone loss and probable need for additional surgery.

**Figure 2:**
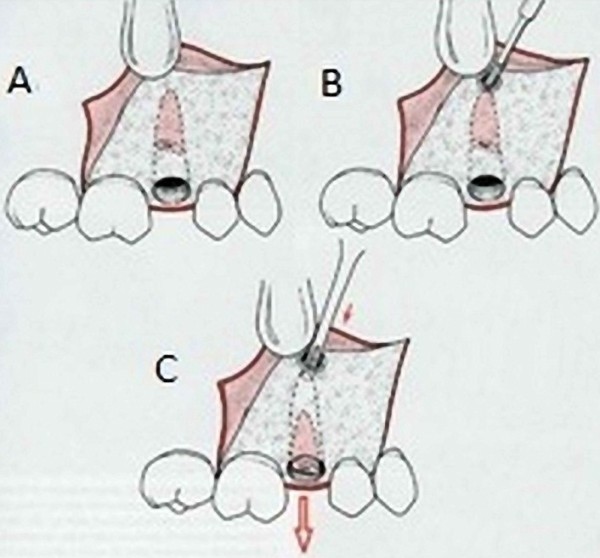
**Open-window approach for root is indicated when buccocrestal bone must be maintained. A**) three-cornered flap is reflected to expose area overlying apex of root fragment being recovered, **B**) bur is used to uncover apex of root and allow sufficient access for insertion of straight elevator and **C**) small straight elevator is then used to displace root out of its socket [1].

Retrieving broken teeth roots is generally accompanied with invasive techniques as mucoperiosteal flaps and removal of buccal bone (Figure 1) or open-window approach (Figure 2) [1]. Consequently, such invasive surgeries are accompanied with post-operative maladies as pain, swelling, trismus, infection, prolonged bleeding, sinus exposure, nerve injury and innervations disorders [13-16,18]. However, the extent of such maladies depend on a number of factors such as the duration of the operation, difficulty of the surgery, the magnitude of the osteotomy/mucoperiosteal flap [7,8], the lack of oral hygiene and the experience of the surgeon [6,19-25].

### Hypothesis

As a non-invasive treatment, EMB has not ever been reported to be utilized in dental surgery. The hypothesis I propose here is that EMB may be an adjunct treatment for extractions of broken teeth roots. This hypothesis is based on the following points: (1) No need for mucoperiosteal flap, hence preservation of soft tissue, (2) no need for osteotomy, hence retention of buccal bone, (3) reduced invasion to surrounding anatomical structures and less risk of sinus exposure, hence control of operative and post-operative complications.

### Proposed parts of EMB

**Figure 3:**
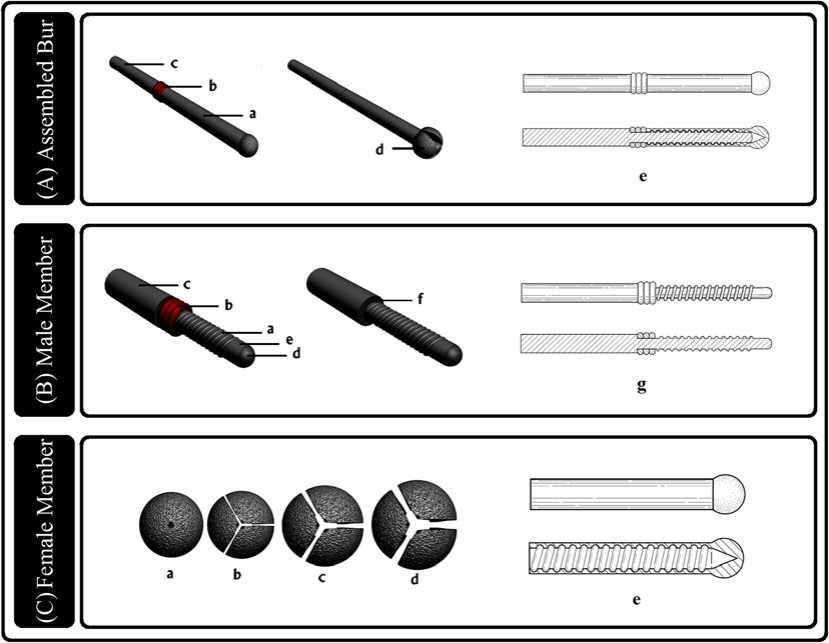
**Structural components of Expandable Micro-motor Bur. A)** Bur components in position, Bur Head (female member) (a), Spacers (b), Bur Base (male member) (c) clutched together for mounting to micro-motor through Main shank, expanded Bur Head (d) and cross-section view (e). **B**) Bur Base (male member) consists of Spiral grooved shaft (a), Spacers (b) and Main shank (c); the tip portion is round (d) and grooveless (e), spacers removed from male member (f) and cross-section view (g). **C**) Bur Head (female member), tip view of Bur Head spinning in root canal. Different degrees of expansion occurs in the canal as shown while Spiral grooved shaft bears three (a), two (b), one (c), no spacers (d) and cross-section view (e).

As indicated in Figure 3, structural components of EMB include: 1) Bur Head which consists of Split round bur and Hallow grooved shaft and 2) Bur base which consists of Spiral grooved shaft, Spacers and Main shank. A micro motor would power its spin.

### Clinical implication

**Figure 4:**
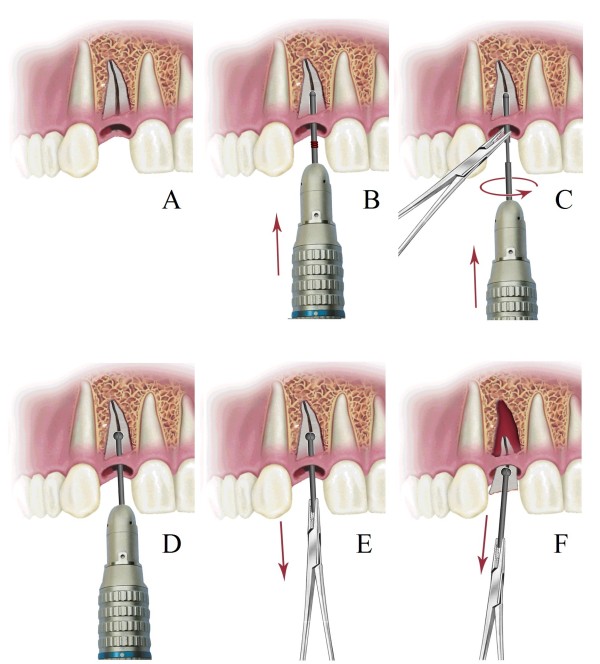
**Proposed procedure in which Expandable Micro-motor Bur removes broken root from its socket. A**) Broken root in its socket. **B**) By using micro-motor, Round bur drilled about half way through root canal similar to a conventional bur. Note that spacers are on. **C**) Spin stops, EMB removed from canal, micro motor detached from EMB, Round bur unscrewed from Bur base, Spacers reduced or totally removed, Round bur placed back into canal, Bur base mounted on micro-motor, by help of a needle holder Round bur and Bur base screwed back. Expanding Round bur carves its periphery making a spherical cavity around itself. This step is run for a very short period. **D**) Spin stops, Micro-motor detached and EMB remains in place. While expanded in root canal, Split round bur behaves as an efficient extraction aiding anchor. **E**, **F**) The firm anchor eases extraction of the broken root with the help of a needle holder.

EMB is proposed to remove broken teeth roots in a conservative manner. It bears less stress for both patient and practitioner. Details of the implementation of EMB to remove a broken tooth root are schematically presented stepwise in Figure 4. The following points support EMB’s applicability for clinical use: 1) with expansion in root canal it behaves as an efficient extraction aiding anchor (as a false tooth crown), 2) since expansion and carving occur simultaneously in cavity (unlike expansion of a screw anchor in a wall hole), cracking in root walls is prevented, 3) there is low chance for breakage of the root walls since the carved cavity made by EMB is spherical and formation of sharp edges is prevented, hence the force applied for extraction will be evenly distributed in the root, 4) it is suitable for all sizes of roots since the expansion rate of the Split round bur is adjusted with application of suitable bur sizes and also use of proper number of spacers. Accordingly, for smaller root, there would be need for smaller bur size and less reduction in spacer numbers (causing minute expansion), 5) Bur Head is disposable single use, hence low chance of infection, 6) minimum post-extraction disorders, 7) shortened rehabilitation period, and 8) freshly evacuated root sockets are useful in dental implants for restoration in patients with tooth loss upon which a higher survival rate of the implants will ensue [3,17]. Comparison between conventional complicated root-extraction with EMB procedures is presented in Table 1.

**Table 1:** Comparison between "conventional extraction of complicated roots "and "Expandable Micro-motor Bur technique"

**Criteria**	**Conventional complicated root-extraction**	**Expandable Micro-motor Bur**
Invasion to surrounding anatomical structures (maxillary sinus exposure, nerve injury and innervations disorders)	More probable	Less probable
Need for mucoperiosteal flap and/or osteotomy of buccal bone	More probable	No or with less extension
Surgical approach	More invasive	More conservative
Operative and post-operative complications	More probable	Less probable
Operation duration	Longer	Shorter
Stress of practitioner and patient	More	Less
Success of immediate implant placement	Less probable	More probable

### Future testing

Considering the application of this treatment, further studies are needed to confirm its effect in clinical cases. The effectiveness of EMB should be verified firstly by animal experiments. The likelihood of its negative influence on nearby vascular and nerve system should be well evaluated. When these concerns are clear, I believe that EMB could be used as a new tool to assist removal of broken teeth roots in humans.

## Competing interest

The author declares that he has no competing interests with others.

I also declare that I have no financial and personal relationships with other people or organizations that can inappropriately influence my work, there is no professional or other personal interest of any nature or kind in any product, service and/or company that could be construed as influencing the position presented in, the article entitled, “Expandable micro-motor bur, design of a new device for least invasive extraction of broken teeth roots”.

## Authors’ contributions

AHSB carried out the entire design of the study and draft the manuscript. He read and approved the final manuscript.
